# Predicting protein-ligand binding residues with deep convolutional neural networks

**DOI:** 10.1186/s12859-019-2672-1

**Published:** 2019-02-26

**Authors:** Yifeng Cui, Qiwen Dong, Daocheng Hong, Xikun Wang

**Affiliations:** 1 Faculty of Education, East China Normal University, 3663 N. Zhongshan Rd., Shanghai, 200062 China; 2 School of Data Science & Engineering, East China Normal University, Shanghai, 3663 N. Zhongshan Rd., Shanghai, 200062 China; 3 The High School Affiliated of Liaoning Normal University, Dalian, China

**Keywords:** Protein, Binding residues, Sequence-based methods, 3D-structure-based methods, Deep convolutional networks

## Abstract

**Background:**

Ligand-binding proteins play key roles in many biological processes. Identification of protein-ligand binding residues is important in understanding the biological functions of proteins. Existing computational methods can be roughly categorized as sequence-based or 3D-structure-based methods. All these methods are based on traditional machine learning. In a series of binding residue prediction tasks, 3D-structure-based methods are widely superior to sequence-based methods. However, due to the great number of proteins with known amino acid sequences, sequence-based methods have considerable room for improvement with the development of deep learning. Therefore, prediction of protein-ligand binding residues with deep learning requires study.

**Results:**

In this study, we propose a new sequence-based approach called DeepCSeqSite for *ab initio* protein-ligand binding residue prediction. DeepCSeqSite includes a standard edition and an enhanced edition. The classifier of DeepCSeqSite is based on a deep convolutional neural network. Several convolutional layers are stacked on top of each other to extract hierarchical features. The size of the effective context scope is expanded as the number of convolutional layers increases. The long-distance dependencies between residues can be captured by the large effective context scope, and stacking several layers enables the maximum length of dependencies to be precisely controlled. The extracted features are ultimately combined through one-by-one convolution kernels and softmax to predict whether the residues are binding residues. The state-of-the-art ligand-binding method COACH and some of its submethods are selected as baselines. The methods are tested on a set of 151 nonredundant proteins and three extended test sets. Experiments show that the improvement of the Matthews correlation coefficient (MCC) is no less than 0.05. In addition, a training data augmentation method that slightly improves the performance is discussed in this study.

**Conclusions:**

Without using any templates that include 3D-structure data, DeepCSeqSite significantlyoutperforms existing sequence-based and 3D-structure-based methods, including COACH. Augmentation of the training sets slightly improves the performance. The model, code and datasets are available at https://github.com/yfCuiFaith/DeepCSeqSite.

## Background

Benefiting from the development of massive signature sequencing, protein sequencing is becoming faster and less expensive. By contrast, owing to the technical difficulties and high cost of experimental determination, the structural details of only small parts of proteins are known in terms of protein-ligand interaction. Both biological and therapeutic studies require accurate computational methods for predicting protein-ligand binding residues [1].

The primary structure of a protein directly determines the tertiary structure, and the binding residues of proteins are closely bound with the tertiary structure. These properties of proteins ensure the feasibility of predicting binding residues from amino acid sequences (primary structures) or 3D structures. However, the complex relationship between binding residues and structures is not completely clear. Thus, we have motivation for using machine learning in binding residue prediction, which is based on the unknown complex mappings from structures to binding residues.

The existing methods for computational prediction of protein-ligand binding residues can be roughly categorized as sequence-based [2–5] or 3D-structure-based methods [1, 6–11]. The fundamental difference between the two types of methods is whether 3D-structure data are used. Some consensus approaches comprehensively consider the results of several methods. These methods can be seen as 3D-structure-based methods if any submethod uses 3D-structure data. Up to now, 3D-structure-based methods have been shown to be widely superior to sequence-based methods in a series of binding residue prediction tasks [1, 11]. However, 3D-structure-based methods depend on a large number of 3D-structure templates for matching. The time cost of template matching for a protein can reach several hours in a distributed environment. Furthermore, the number of proteins with known amino acid sequence is three orders of magnitude higher than that of proteins with known 3D structures. The enormous disparity in these quantities leads to difficulties in effectively utilizing 3D-structure information and massive sequence information together, which limits further progress in binding residue prediction.

A series of traditional machine learning methods have been used in binding residue prediction. Many computational methods based on support vector machines (SVM) have been proposed for specific types of binding residue prediction [12–15]. A traditional BP neural network has been used in protein-metal binding residue prediction, but the network has considerable room for improvement [16]. Differing in interpretability from the mentioned methods, a robust method based on a Bayesian classifier has been developed for zinc-binding residue prediction [17]. Many methods based on template matching achieve considerable success at the expense of massive computational complexity [1, 10, 11]. A representative consensus approach, COACH, combines the prediction results of TM-SITE, S-SITE, COFACTOR, FINDSITE and ConCavity, some of which are 3D-structure-based methods [1, 6, 7, 10, 11]. This robust approach to protein-ligand binding residue recognition substantially improves the Matthews correlation coefficient (MCC). These methods have achieved successful results on small datasets. However, the methods would achieve even higher accuracy if massive data could be further utilized. One crucial factor for the available utilization of massive data is the representation capability of classifiers, which has a dominant impact on generalization.

Deep neural networks have achieved a series of breakthroughs in image classification, natural language processing and many other fields [18–21]. In bioinformatics, deep neural networks have been applied in many tasks, including RNA-protein binding residue prediction, protein secondary structure prediction, compound-protein interaction prediction and protein contact map prediction [22–25]. Various recurrent networks are commonly used in sequence modeling [26, 27]. Context dependencies universally existing in sequences can be captured effectively by recurrent networks, and these networks are naturally suitable for variable-length sequences. Nevertheless, recurrent networks depend on the computations of the previous time step, which blocks parallel computing within a sequence. To solve this problem, convolutional neural networks are introduced into neural machine translation (NMT) [28, 29]. These architectures are called temporal convolution networks (TCN). In contrast to recurrent networks, the computation within a convolutional layer does not depend on the computation of the previous time step, so the calculation of each part is independent and can be parallelized. Convolutional sequence-to-sequence models outperform mature recurrent models on very large benchmark datasets by an order of magnitude in terms of speed and have achieved the state-of-the-art results on several public benchmark datasets [29]. Many similarities exist between NMT and binding residue prediction. The performance of binding residue prediction can be improved with progress in NMT.

In this study, we propose a new approach, DeepCSeqSite (DCS-SI), for protein-ligand binding residue prediction. The architecture of DCS-SI is inspired by a series of sequence-to-sequence models including ConvS2SNet [29]. DCS-SI includes two editions: stdDCS-SI and enDCS-SI. The encoders of the two editions are the same. The decoder of enDCS-SI evolves from the decoder of stdDCS-SI. The former executes forward propagation twice and takes the previous output into consideration to produce more accurate predictions. In DCS-SI, the fully convolutional architecture contributes to improving parallelism and processing variable-length inputs. Several convolutional layers are stacked on top of each other to extract hierarchical features. The low-level features reflect local information over residues near the target while the high-level features reflect global information over a long range of an amino acid sequence. Correspondingly, the size of the effective context scope is expanded as the number of layers increases. The long-distance dependencies between the residues can be captured by an effective context scope that is sufficiently large. A simple gating mechanism is adopted to select relevant residues. Templates are not used in DCS-SI. The network in DCS-SI is trained only on sequence information. The state-of-the-art ligand-binding method COACH and some of its submethods are selected as baselines. Experiments show that stdDCS-SI and enDCS-SI significantly outperform the baselines.

## Methods

### Datasets

The datasets used in this study are collected from the BioLip database and the previous benchmarks [1, 11]. Our training sets contain binding residues of fourteen ligands (ADP, ATP, Ca ^2+^, Fe ^3+^, FMN, GDP, HEM, Mg ^2+^, Mn ^2+^, Na ^+^, NAD, PO$_{4}^{3-}$, SO$_{4}^{2-}$, Zn ^2+^)^1^. A total of 151 proteins are selected from the previous benchmarks with the fourteen ligands as the benchmark testing set, called SITA. Every protein in the training sets has a sequence identity to the proteins in the validation sets and testing sets of less than 40% [13]. To obtain as much data as possible for training, the pairwise sequence identity is allowed to be 100% in the training sets. We speculate that the augmented training sets (Aug-Train) can drive networks to achieve better generalization performance.

Considerable data skew generally exists in protein-ligand binding residue prediction. ADP, ATP, FMN, GDP, HEM and NAD have more binding residues than do metal ions and acid radical ions, which means that the substantial data skew is attributed more to metal ions and acid radical ions. The computational binding residue prediction of metal ions and acid radical ions is still difficult because of the small size and high versatility. To demonstrate the ability of the models to predict the binding residues of metal ions and acid radical ions, we extend SITA with metal ions and acid radical ions. Every protein in the testing sets has a sequence identity to the proteins in the training sets and the other testing sets of less than 40%. Furthermore, the extended testing sets (SITA-EX1, SITA-EX2 and SITA-EX3) reduce the variance in the tests.

A summary of the datasets used in this study is shown in Table 1. Severe data skew exists in the datasets, which restricts the optimization and performance of many machine learning algorithms. The data skew is considered in the design of DCS-SI.

**Table 1:** Summary of the datasets

	$\phantom {\dot {i}\!}N_{Prot^{1}}$	$\phantom {\dot {i}\!}N_{BR^{2}}$	$\phantom {\dot {i}\!}N_{NBR^{3}}$	$\phantom {\dot {i}\!}P_{BR(\%)^{4}}$
Train ^5^	4237	34564	1245517	2.78
Aug-Train	37821	348180	11310574	3.08
SITA	151	1340	45126	2.97
SITA-EX1	187	1528	56122	2.72
SITA-EX2	226	1725	68729	2.51
SITA-EX3	253	1890	75161	2.51

^1^ *N*_*Prot*_: number of proteins

^2^ *N*_*BR*_: number of binding residues

^3^ *N*_*NBR*_: number of non-binding residues

^4^ *P*_*BR*_: proportion of binding residues

^5^Train: original training set

### Method motivation

Each residue in an amino acid sequence plays a specific role in the structure and function of a protein. For a target residue, nearby residues in the tertiary structure plausibly affect whether the target residue is a binding residue for some ligand. Thus, residues near the target residue in the tertiary structure but far from the target residue in the primary structure are critical to binding residue prediction. Most of the existing methods use a sliding window centered at the target residue to generate overlapping segments for every target protein sequence [13, 16, 30]. The use of sliding windows is a key point in converting several variable-length inputs into segments of equal length. However, even if the distance in the sequence between two residues is very long, their spatial distance can be limited because of protein folding. Thus, residues far from the target residue in the sequence may also have an important impact on the location of the binding residues. To obtain more information, these methods have to increase the window size in the data preprocessing stage. The cost of computation and memory for segmentation is not acceptable when the window size increases to a certain width.

On the basis of the inspiration from NMT, protein-ligand binding residue prediction can be seen as a particular form of translation. The main differences are the following two aspects: 1. For NMT, the elements in the destination sequences are peer entities to the elements in the source sequences, but the binding site labels are not peer entities to the residues. 2. While the destination and source sequences typically differ in length for NMT, a one-to-one match between each binding residue label and each residue exists. Despite the differences, binding residue prediction can learn from NMT. The foundation of feature extraction in NMT includes local correlation and long-distance dependency, which are common in amino acid sequences and natural language sentences. Thus, the main idea of feature extraction in NMT is applicable to binding residue prediction.

### Method outline

**Fig. 1:**
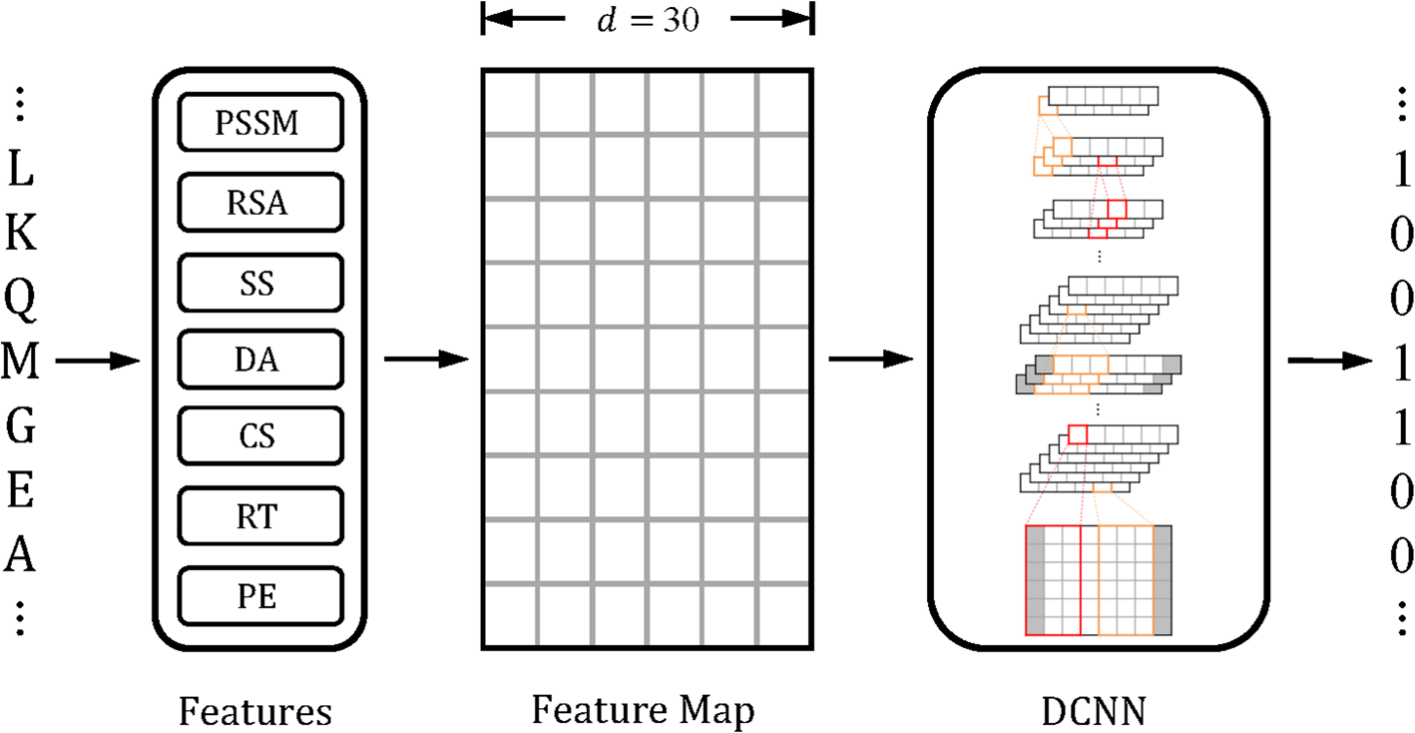
Method Outline. Each residue in the amino acid sequence is embedded in a feature space that consists of seven types of features, namely, position-specific score matrix (PSSM), relative solvent accessibility (RSA), secondary structures (SS), dihedral angle (DA), conservation scores (CS), residue type (RT) and position embeddings (PE). The dimension number *d* of the feature space is 30. The amino acid sequence is transformed into a feature map as the input for the deep convolutional neural network, which outputs the result of the protein-ligand binding residue prediction. Each cell represents a dimension of the feature map

In the training sets, the binding residues that belong to any selected ligand type are labeled as positive samples, and the rest are labeled as negative samples. A deep convolutional neural network is trained as the classifier of stdDCS-SI or enDCS-SI, whose inputs are entire amino acid sequences. The input sequences are allowed to differ in length. The sequences are divided into several batches during training. In each batch, the sequences are padded to the length of the longest sequence in the batch with dummy residues. Batches are allowed to differ in length after padding. Each protein residue is embedded in a feature space consisting of several features to construct the input feature map for the classifier. For a given protein, every residue is predicted to be a binding residue or non-binding residue in the range of the selected ligand types simultaneously. The representation of dummy residues is removed immediately before the softmax layer. The method outline is shown in Fig. 1. The details of the method are described in “Architecture” section.

### Features

Seven types of features are used for the protein-ligand binding residue prediction: position-specific score matrix (PSSM), relative solvent accessibility (RSA), secondary structure (SS), dihedral angle (DA), conservation scores (CS), residue type (RT) and position embeddings (PE).

#### PSSM

PSSM is the probability of mutating to each type of amino acid at each position. Therefore, PSSM can be interpreted as representing conservation information. Normalized PSSM scores can be calculated as follows:

1 $$ y = \frac{1}{1 + 2^{-x}}  $$

where *x* is the dimension of the PSSM score and *y* is the corresponding PSSM feature. For a protein with *L* residues, the PSSM feature dimension is *L*∗20.

#### Relative solvent accessibility

The RSA is predicted by SOLVE. The real value of RSA is generally converted to a Boolean value indicating whether the residue is buried (RSA <25%) or exposed (RSA >25%). However, the original value is retained so that the network in DCS-SI can learn more abundant features [31].

#### Secondary structure

The secondary structure is predicted by PSSpred. The secondary structure type (alpha-helix, beta-strand and coil) is represented by a real 3D value. Each dimension of the real 3D value is in the range of [0, 1] indicating the possibility of existence of the corresponding type [32].

#### Dihedral angle

A real 2D value specifying the *ϕ*/ *ψ* dihedral angles is predicted by ANGLOR [33]. The values of *ϕ* and *ψ* are normalized by *Norm*(*x*)=*x*/360.0.

#### Conservation scores

Conservation analysis is a widely used method for detecting ligand-binding residues [34, 35]. Ligand-binding residues tend to be conserved in evolution because of their functional importance [2]. The relative entropy (RE) and Jensen-Shannon divergence (JSD) scores of conservation are taken as features in this study.

#### Residue type

Some amino acids have a much higher binding frequency for the corresponding ligands than do other amino acids. Twenty amino acid residues and an additional dummy residue are numbered from 0 to 20. Then, the numbers representing residue type are restricted to the range of [0, 1] by dividing by the total number of the types.

#### Position embeddings

Position embeddings can carry information about the relative or absolute position of the tokens in a sequence [36]. Several methods have been proposed for position embeddings. Experiments with ConvS2SNet and Transformer show that position embeddings can slightly improve performance, but the difference among several position embedding methods is not clear [29, 36]. Therefore, a simple method for position embeddings is adopted in DCS-SI. The absolute positions of the residues are represented as *P* *E*_*i*_=*i*/*L*, where *P* *E*_*i*_ of the *i*-th residue is limited to range [0, 1], and *L* is the length of the amino acid sequence.

### Architecture

The effective context scope for the prediction result or hidden layer representation of a target residue is called the input field. The size of the input field is determined by the stacked convolutional layers instead of being explicitly specified. Stacking *n* convolutional layers with kernel width *k* and stride =1 results in an input field of 1+*n*(*k*−1) elements (including padded elements). The input field can easily be enlarged by stacking more layers, which enables the maximum length of the dependencies to be precisely controlled. The stacked convolutional layers have the ability to process variable-length input without segmentation, which significantly reduces the additional cost. Moreover, deeper networks can be constructed with the slow growth of parameters. However, many proteins have hundreds or even thousands of residues; thus, deep stacked convolutional layers or a very large kernel width is required for long-distance dependencies. The latter is unadvisable because padded elements in the input fields and the growth rate of parameters are incremental over kernel width. By contrast, going deeper enables the method to achieve the desired results.

#### stdDCS-SI

**Fig. 2:**
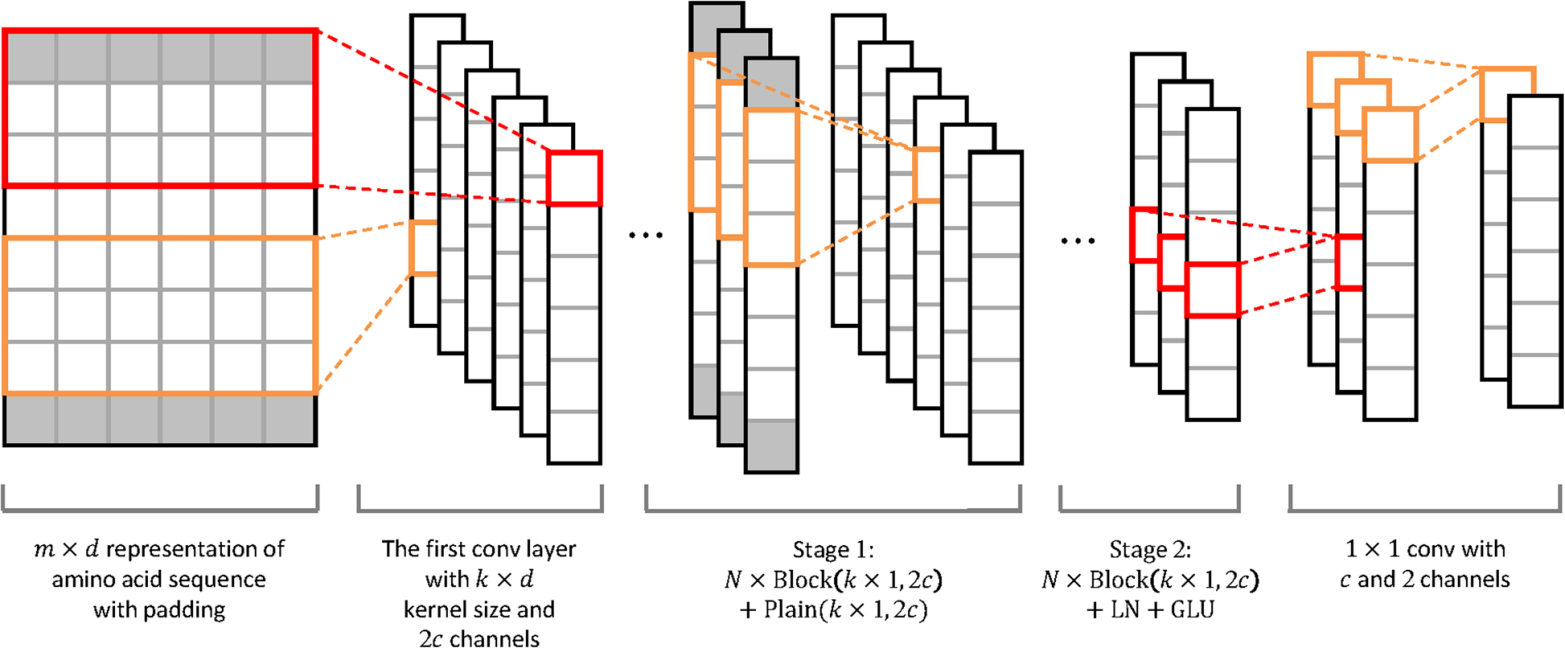
Architecture of the deep convolutional neural network in std-DeepCSeqSite (stdDCS-SI). Each cell represents a dimension of a representation. The *m*×*d* representation of an amino acid sequence is the input of the network, where *m* is the length of the amino acid sequence, and *d* is the dimension number of the feature space. Block (*k*×1,2*c*) represents a BasicBlock with a *k*×1 kernel size and 2*c* output channels, and the structure of Plain (*k*×1,2*c*) is the same as that of Block (*k*×1,2*c*) without residual connection. The situation of *k*=3, stride =1 and *c*=3 is described in this figure. Each *m*×1 cell grid represents the output of a convolution kernel. The right-most representation is the input for the softmax

The architecture of the deep convolutional neural network is shown in Fig. 2. The input to the network consists of *m* residues embedded in *d* dimensions. Due to the local correlation among the representations of adjacent residues, 1D convolution along the sequence is applied to the initial feature map and the hidden feature maps. The local correlation is based on the interaction among nearby residues and the covalent bond between adjacent residues.

For the encoder network, each residue always has a representation during forward propagation. A group of *k*×*d* convolution kernels transforms the initial *m*×*d* feature map into *m*×1×2*c*, where 2*c* is the output channel number of the convolution kernels. Zero elements are padded at both sides of the initial feature map to maintain *m*. The transformation and padding aim to satisfy the input demands of the following layers and the feature extraction. The main process of the network can be separated into two stages. Each stage contains *N* BasicBlocks (described in “BasicBlock” section) that consist of multiple frequently used layers and are designed for cohesiveness and expandability. In each stage, blocks are stacked on top of each other to learn hierarchical features from the input of the bottom block. At the tops of each stage, additional layers are added to stabilize the gradients and normalize the outputs.

For the decoder network, the representation of each residue is transformed into the distribution over possible labels. Following the two stages, two fully connected layers consisting of one-by-one (1×1) convolution kernels are used for information interaction between channels. The numbers of output channels of these 1×1 convolution kernels are set to *c* and 2. The number of elements represented by the output of each block or layer is the same as the number of initial input elements. The first fully connected layer is wrapped in dropout to prevent overfitting [37]. The output of the last fully connected layer is fed to a 2-way softmax classifier, which produces the distribution over the labels of positive and negative samples.

The cross entropy between the training data distribution and the model distribution is used in the following cost function:

2 $$ J(\theta)=-\sum\limits_{i}^{t}\widehat{P}\left(y^{(i)}|x^{(i)}\right)\log{P\left(y^{(i)}|x^{(i)};\theta\right)}+\gamma\cdot\|\theta\|_{2}^{2}  $$

where *θ* represents the weights in DCS-SI, {*x*^(1)^,⋯,*x*^(*t*)^} is a set of *t* samples, {*y*^(1)^,⋯,*y*^(*t*)^} is a set of corresponding labels (*y*^(*i*)^∈{0,1}) and *γ* is the coefficient of the L2 normalization $\|\theta \|_{2}^{2}$.

#### enDCS-SI

We proposed enDCS-SI on the basis of stdDCS-SI. Note that the prediction of the other residues is called the context prediction. Although stdDCS-SI outperforms existing methods, the performance can be further improved if the context prediction is taken into consideration explicitly. To achieve this goal, we retained the encoder network and modified the decoder network. In addition to the output of the encoder network, the new decoder network receives the context prediction as input. A group of *k*×2 convolution kernels transforms the context prediction into *m*×1×2*c*, where 2*c* is the number of output channels of the convolution kernels. The following process consists of two parallel stages with *M* blocks and additional layers (in this study, we use *M*=2). To extract the features from the left (right) context prediction, we remove 1 element from the end (start) of the context prediction. Then, the input of each convolutional layer is padded by *k* elements on the left (right) side. The extracted information of the left and right adjacent predictions is directly added to the output of the encoder, where the three tensors have the same shape. ConvS2SNet directly uses the labels as the context prediction during training. Therefore, the forward propagation in training operates in parallel with the sequence. However, no label exists for the input samples during testing. Thus, the prediction for each element is processed serially to generate the context prediction for the next element.

**Fig. 3:**
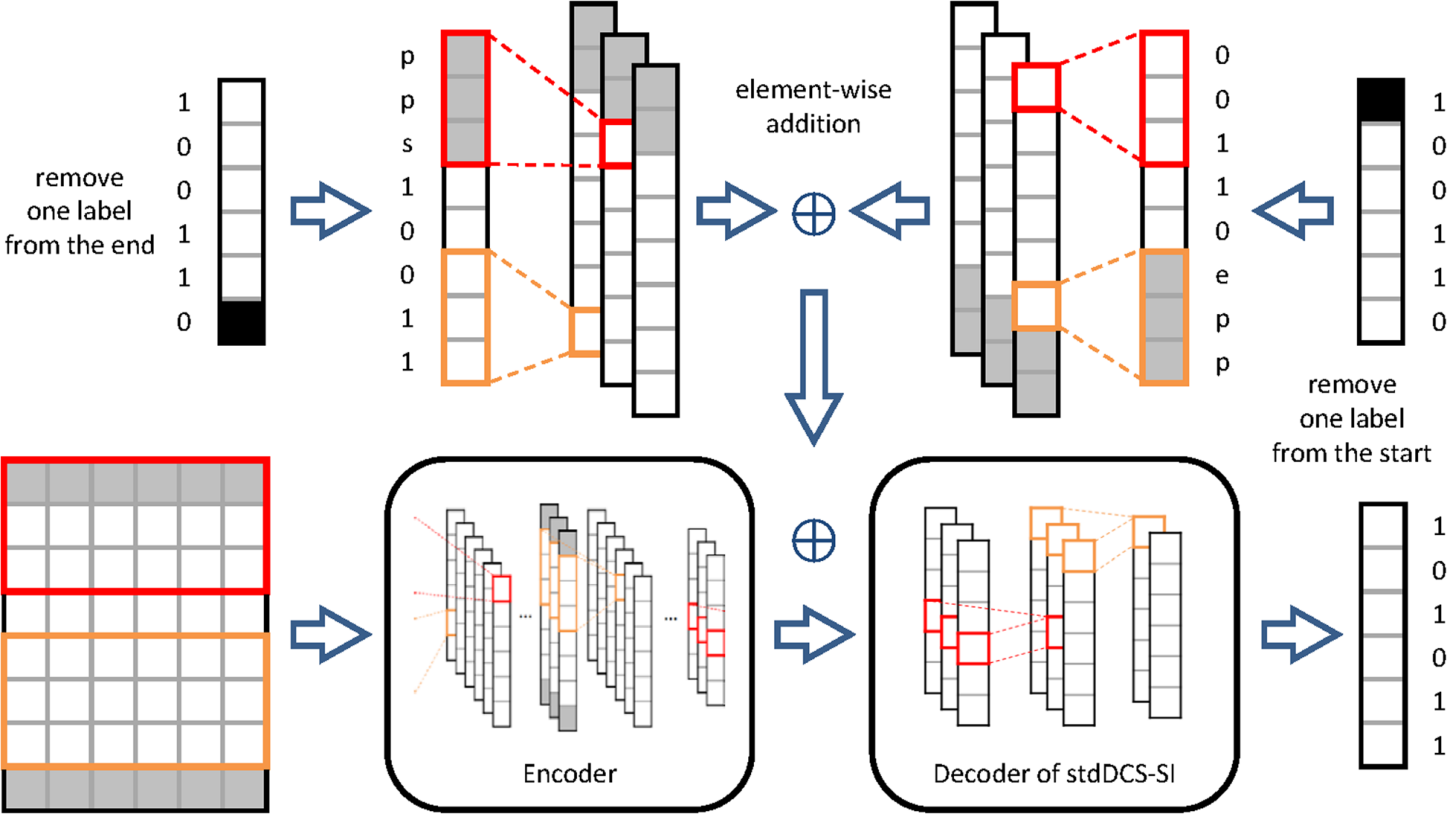
Architecture of the deep convolutional neural network in en-DeepCSeqSite (enDCS-SI). The encoder of enDCS-SI is the same as that of stdDCS-SI. The decoder of enDCS-SI is designed to extract the information form the labels or the previous prediction. The decoder of stdDCS-SI is included in the decoder of enDCS-SI, where the weights of the former are fine-tuned during training enDCS-SI. ‘p’, ‘s’ and ‘e’ represent padding, start mark and end mark

To overcome the serialization in testing, we let enDCS-SI execute forward propagation in the decoder network 2 times. The first forward propagation is similar to that of stdDCS-SI, but the context prediction for enDCS-SI is fed by a zero tensor. The output of the first forward propagation is used as the context prediction for enDCS-SI in the second forward propagation. While training enDCS-SI, the context prediction is also replaced with the labels. All the weights in stdDCS-SI are loaded for enDCS-SI. The rest of the weights of enDCS-SI are initialized. The weights of the encoder network are fixed because the encoding processes of stdDCS-SI and enDCS-SI are the same. The architecture of enDCS-SI is described in Fig. 3.

#### BasicBlock

The input of BasicBlock is processed in the order LN-GLU-Conv. The output of the *l*-th block is designated as $\textbf {s}^{l}=\left (s^{1},..., s^{m}\right) \in \mathbb {R}^{m \times 1 \times 2c}$, where *m* is the length of the input sequences^2^ and *c* is the number of input channels of convolutional layer in each block. The output of the *l* − 1-th block is input to the *l*-th block. The input of each *k*×1 convolution kernel is an *m*×1×*c* feature map consisting of *m* input elements mapped to *c* channels. Before convolution, both ends of each channel are zero-padded with *k*/2 elements to maintain the height of the feature map, where the height is *m*. A convolutional layer with 2*c* output channels transforms the input of convolution $X \in \mathbb {R}^{m \times 1 \times c}$ into the output of convolution $Y \in \mathbb {R}^{m \times 1 \times 2c}$ to satisfy the input requirement of the gated linear units (GLU) of the next possible block and to make the input size and output size of the block consistent [38]. *Y* corresponds to $[A\ B] \in \mathbb {R}^{m \times 1 \times 2c}$, where $A, B \in \mathbb {R}^{m \times 1 \times c}$ are the inputs to the GLU. A simple gating mechanism over [*A* *B*] is implemented as follows:

3 $$ g([A\ B]\!) = A \otimes \sigma(B)  $$

where *σ* represents the sigmoid function. The output of GLU $g([A\ B]\!) \in \mathbb {R}^{m \times 1 \times c}$ is one-half the size of Y and is the same as the input size of the convolution in BasicBlock.

**Fig. 4:**
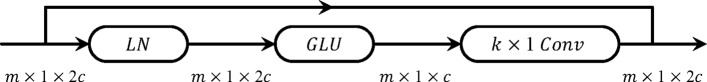
Architecture of BasicBlock. The input of a BasicBlock is processed in the order LN-GLU-Conv. The output of a BasicBlock is the sum of the input and the Conv output. The shapes of the input/output for each layer in a BasicBlock are shown in the figure, where *m* is the length of the amino acid sequence and 2*c* is the number of output channels of the BasicBlock

GLU can select the relevant context for the target residue by the means of activated gating unit *σ*(*B*). The gradient of GLU has a path that without downscaling contributes to the flow of the gradient, which is an important reason for the choice of the activation function. The vanishing gradient problem is considered before going deeper. Hence, residual connections from the input of the block to the output of the block are introduced to prevent the vanishing gradient [20]. The input of a block must be normalized before convolution because the input is the sum of the outputs of the several previous blocks. Without normalization, gradients are unexpected during training. Therefore, a LayerNormalization (LN) layer is set at the beginning of the block to provide a stable gradient, which is also conductive to accelerating the learning speed [39]. The function of BasicBlock is summarized in Eq.(4):

4 $$ s^{l}_{i} = W^{l}\left(GLU\left[s^{l - 1}_{i - k / 2},..., s^{l-1}_{i + k / 2}\right]_{L\!N}\right) + s^{l - 1}_{i}   $$

where *W*^*l*^ represents the weights of convolution in the *l*-th block, $s^{l}_{i}$ is the features of the *i*-th element represented in the *l*-th block, *k* is the width of the convolution kernels and subscript *L* *N* means that $\left [s^{l - 1}_{i - k / 2},..., s^{l-1}_{i + k / 2}\right ]$ has been normalized by LN. The details are described in Fig. 4.

### Evaluation

The main evaluation metrics for binding residue prediction results include the Matthews correlation coefficient (MCC), precision (%) and recall (%), which are defined as follows:

5 $$\hspace{12pt} MCC\,=\,\frac{T\!P\!\times\!T\!N\,-\,F\!P\!\times\!F\!N}{\sqrt{(T\!P\,+\,F\!P)(T\!P\,+\,F\!N)(T\!N\,+\,F\!P)(T\!N\,+\,F\!N)}}  $$

6 $$ precision = \frac{T\!P}{T\!P\,+\,F\!P}  $$

7 $$\hspace{10pt} recall = \frac{T\!P}{T\!P\,+\,F\!N}  $$

where *T* *P* is the number of binding residues predicted correctly, *F* *P* is the number of non-binding residues predicted as binding residues, *T* *N* is the number of non-binding residues predicted correctly and *F* *N* is the number of binding residues predicted as non-binding residues.

## Results

### Optimization

For the hyperparameter choice, we focus on the number of BasicBlocks *N* and the kernel width *k* in the BasicBlocks. *N* and *k* both have a decisive effect on the parameter space and the maximum length of the dependencies. Thus, *N* and *k* are closely related to the generalization and are separately adjusted to obtain the local optimum. When adjusting *N*, the kernel size of each BasicBlock is fixed to 3×1 (*k*=3). When adjusting *k*, *N* is fixed to 10. The output channel number of each BasicBlock is set to 512 (*c*=256) in this study. Experiments show that the network achieves the locally optimal generalization on the validation sets when *N*=10 and *k*=5^3^. The details are shown in Tables 2 and 3.

**Table 2:** The effect of depth on the validation sets

	*N*=2	*N*=4	*N*=6	*N*=8	*N*=10	*N*=12	*N*=14
MCC	0.422	0.441	0.436	0.458	0.482	0.475	0.451
Precision	45.07	50.87	48.24	52.66	57.87	58.33	53.80
Recall	42.37	40.55	42.07	42.11	42.11	40.60	39.92

**Table 3:** The effect of kernel width on the validation sets

	*k*=3	*k*=5	*k*=7	*k*=9
MCC	0.482	0.495	0.485	0.479
Precision	57.87	59.85	58.91	58.20
Recall	42.11	42.85	41.91	41.31

Experiments indicate that DCS-SI can be optimized effectively on the training sets and achieve good generalization on the test sets without any sampling. Mini-batches are prone to contain only negative samples if the samples are grouped via inappropriate methods. This problem is unlikely to occur in our mini-batches because an amino acid sequence is treated as a unit during our grouping. The severe data skew can be overcome as long as the proportion of positive samples in every mini-batch is close to the actual level. The cost function is minimized through mini-batch gradient descent. With zero-padding, the feature maps of the proteins in a batch are filled to the same size to simplify the programming implementation. The coefficient *γ* of the *L*2-Norm is 0.2, and the dropout ratio is set to 0.5. All DCS-SI models are implemented with TensorFlow. The training process consists of three learning strategies to suit different training stages. The learning rate of each stage decreases exponentially after the specified number of iterations. The gradient may be very steep in the early stage because of the unpredictable error surface and weight initialization. Hence, to preheat the network, the initial learning rate of the first stage is set to a value that can adapt to a steep gradient. Due to the considerable data skew, the training algorithm tends to fall into a local minimum where the network predicts all inputs as negative examples. A conservative learning rate is not sufficient to escape from this type of local minimum. Therefore, the initial learning rate of the second stage can be increased appropriately to search better minimums and further reduce the time cost of training. A robust strategy is required at the end of training to avoid the strong sway phenomenon. The details of the learning strategies are available in our software package.

### The effect of the softmax threshold

DCS-SI tends to predict residues as non-binding residues because the proportion of positive and negative samples in each batch is maintained at approximately the natural proportion. For the binary classification model, the threshold of positive and negative samples has a nonnegligible impact on performance. As shown in Table 4, despite losing some precision, MCC and recall increase with the decreasing threshold, where the threshold is the minimum probability required for a sample to be predicted as positive. When the threshold =0.4, the MCC achieves local optimization.

**Table 4:** Prediction results on the validation sets with different thresholds

Thr ^1^	Precision	Recall	MCC
0.9	66.59	37.67	0.491
0.8	65.21	38.68	0.492
0.7	64.33	39.50	0.494
0.6	63.55	40.24	0.495
0.5	62.66	40.69	0.494
0.4	61.99	41.43	0.496
0.3	60.96	42.08	0.495
0.2	59.85	42.85	0.495
0.1	58.03	43.93	0.493

^1^Thr: The threshold of the softmax

### Comparison with other methods

stdDCS-SI and the baselines are tested on SITA and three extended testing sets. The existing 3D-structure-based methods within the baselines (TM-SI, COF and COA) outperform the sequence-based method S-SI on the testing sets. stdDCS-SI is far superior to all the baselines. The improvements of MCC and precision are no less than 0.05 and 15%, respectively. One possible reason for the moderate recall of stdDCS-SI is that the low percentage of binding residues in the training sets leads to prudent prediction of stdDCS-SI. Improving the recall of stdDCS-SI is a topic for future research. The details are described in Table 5, where the hyperparameters are locally best adjusted for stdDCS-SI (*k*=5, *N*=10 and threshold =0.4). All the baselines used in the experiments are included in the I-TASSER Suite [31].

**Table 5:** Prediction results for the baselines and stdDCS-SI on the testing sets

TestSet	Evaluation	TM-SI ^1^	S-SI ^2^	COF ^3^	COA ^4^	stdDCS-SI ^5^
SITA	MCC	0.337	0.293	0.411	0.423	0.476
	Precision	32.16	21.93	42.06	32.97	58.64
	Recall	47.24	55.71	49.24	75.20	45.82
SIEX1	MCC	0.313	0.280	0.364	0.391	0.465
	Precision	29.93	21.48	36.90	30.59	56.26
	Recall	43.74	52.49	44.53	69.44	45.01
SIEX2	MCC	0.284	0.267	0.325	0.358	0.452
	Precision	26.64	20.61	32.70	27.90	53.78
	Recall	40.42	50.04	40.20	64.44	44.01
SIEX3	MCC	0.278	0.263	0.315	0.343	0.449
	Precision	26.44	20.60	31.79	27.07	53.07
	Recall	39.21	48.41	38.70	61.54	43.90

^1^TM-SI: TM-SITE

^2^S-SI: S-SITE

^3^COF: COFACTOR

^4^COA: COACH

^5^DCS-SI: DeepCSeqSite

All the features used in this study are obtained from sequence or evolution information through computational methods. However, noise is introduced by the predictions of some features, including secondary structures and dihedral angles. The performance of stdDCS-SI will improve if these features are more accurate.

### Comparison of stdDCS-SI and enDCS-SI

The residues adjacent to binding residues have a higher probability of binding than do the other residues. stdDCS-SI does not explicitly consider the aggregation of binding residues. The consideration of aggregation is implicitly included in the transformation of the hidden representation, which is one reason for the good performance of stdDCS-SI. Furthermore, enDCS-SI predicts the binding residues with aggregation explicitly. The decoder network of enDCS-SI can extract useful information from the context prediction. As shown in Table 6, the MCC for each testing set is improved 0.01 ∼ 0.02 by enDCS-SI. Although enDCS-SI requires more time to execute the additional forward propagation in its decoder network, the total time cost of enDCS-SI is not significantly increased. During testing, the predictions for every residue can be executed in parallel. Only the two forward propagations are processed serially. The advantage of enDCS-SI is more prominent if the input amino acid sequences are long and the machines have sufficient computational capacity.

**Table 6:** Prediction results for stdDCS-SI and enDCS-SI

	stdDCS-SI			enDCS-SI		
TestSet	Precision	Recall	MCC	Precision	Recall	MCC
SITA	58.64	45.82	0.476	61.53	47.39	0.498
SIEX1	56.26	45.01	0.465	58.61	45.39	0.478
SIEX2	53.78	44.01	0.452	55.69	44.44	0.462
SIEX3	53.07	43.90	0.449	54.85	44.14	0.456

### The effect of data augmentation

Data augmentation typically contributes to the generalization of deep neural networks. To achieve better generalization, we use redundant proteins to obtain the augmented training sets (Aug-Train). The pairwise sequence identity is allowed to be 100% in Aug-Train, which contains at least nine times as many proteins as the original training sets.

As shown in Table 7, the model trained on Aug-Train has slightly better generalization performance on the testing sets. However, the computational cost has increased several times. Relative to the cost, the improvement from data augmentation is far less than expected. This counterintuitive result indicates that proteins with a high sequence identity contribute little to the generalization of the network. Therefore, data augmentation based on high redundancy is not suitable as the main optimization method in this study.

**Table 7:** The effect of data augmentation

	Train			Aug-Train ^1^		
TestSet ^2^	Precision	Recall	MCC	Precision	Recall	MCC
SITA	56.31	41.55	0.448	58.79	42.27	0.470
SIEX1	53.66	40.20	0.432	55.81	41.45	0.454
SIEX2	50.12	38.80	0.411	53.23	39.79	0.434
SIEX3	49.38	39.07	0.410	52.88	39.81	0.433

^1^Due to the difficulties in training on Aug-Train, networks with *k*=9 and *N*=10 are used in this experiment. The average-cross entropy loss per protein on Train and Aug-Train are 0.80 and 7.77, respectively. The cross-entropy loss on Aug-Train does not change substantially with further training. For fair comparison, we do not use more complex networks

^2^SIEX1: SITA-EX1, SIEX2: SITA-EX2, SIEX3: SITA-EX3

## Discussion

The effective utilization of data contributes to the improvement. Traditional classifiers are used in many existing methods, where the classifiers include SVM and traditional artificial neural networks (ANN). The input features for these classifiers are designed manually, and transformations in these classifiers focus on how to separate the input samples. Further feature extraction is inadequate, which limits the representation and generalization of these classifiers. Deep convolutional neural networks take advantage of massive sequence information. The hierarchical structure has the ability to extract low-level features and to organize low-level features as high-level features. The representation ability of the hierarchical features improves with the increase in layers, which requires sufficient data to ensure generalization. Currently, massive sequence information satisfies this requirement. In addition to the representation ability, the hierarchical structure provides the ability to capture long-distance dependencies. Without segmentation, the maximum distance of dependencies is not limited to the window size. Long-distance dependencies can be reflected in high-level features with a sufficiently large input field.

Most traditional machine learning methods are sensitive to data skew, which fundamentally affects the generalization. The number of binding residues is far less than that of non-binding residues in our datasets, especially for metal ions and acid radical ions. The proportion of binding residues in the datasets is no more than 4%. We have attempted to replace the network in DCS-SI with SVMs. However, SVMs make the normal convergence on the unsampled training sets difficult. Even if the SVMs converge normally, their generalization is challenging. By contrast, the representation of DCS-SI is sufficiently strong to capture effective features for fitting and generalization without sampling. Training without sampling allows the network to learn more valid samples, which also contributes to the improvement.

DCS-SI is better than the baselines in terms of predicting the binding residues of metal ions and acid radical ions. As shown in Table 5, the performance of the baselines decreases when metal ions and acid radical ions are added to SITA. The decrease in MCC is 0.03 ∼ 0.04. Regardless, the performance of DCS-SI remains close to its original level, and the MCC of DCS-SI decreases by no more than 0.02. The contrast indicates that the superiority in predicting the binding residues of metal ions and acid radical ions is a direct source of the improvement.

## Conclusion

We propose a sequence-based method called DeepCSeqSite (DCS-SI), which introduces deep convolutional neural networks for protein-ligand binding residue prediction. The convolutional architecture effectively improves the predictive performance. The highlights from DCS-SI are as follows:

1. The convolutional architecture in DCS-SI provides the ability to process variable-length inputs.
2. The hierarchical structure of the architecture enables DCS-SI to capture the long-distance dependencies between the residues, and the maximum length of the dependencies can be precisely controlled.
3. Augmentation of the training sets slightly improves the performance, but the computational cost for training increases several times.
4. Without using any template including 3D-structure data, DCS-SI significantly outperforms existing sequence-based and 3D-structure-based methods, including COACH.

In future work, we plan to access the residues correlation at long distance by various attention mechanisms. Furthermore, the application of finite 3D-structure data to deep convolutional neural networks may effectively improve the protein-ligand binding residue prediction performance. Generative adversarial nets is a method that is worth applying to attempt to solve the severe deficiency of 3D-structure data relative to sequence data [40].

## Abbreviations

ANN: Artificial neural network
CS: Conservation scores
DA: Dihedral angle
DCS-SI: DeepCSeqSite
enDCS-SI: en-DeepCSeqSite
GLU: Gated linear units
JSD: Jensen-Shannon divergence
MCC: Matthews correlation coefficient
NMT: Neural machine translation
PE: Position embeddings
PSSM: Position-specific scoring matrices
RE: Relative entropy
RSA: Relative solvent accessibility
RT: Residue type
SS: Secondary structures
stdDCS-SI: std-DeepCSeqSite
SVM: Support vector machine
TCN: Temporal convolution network
